# Genistein and Curcumin Inhibit Proliferation and Invasiveness in BRAFV600E Mutant and Wild-Type Melanoma Cells: Insights into Their Anticancer Effects

**DOI:** 10.3390/biomedicines13081954

**Published:** 2025-08-10

**Authors:** Federico Vaccaro, Federica Mannino, Mariacarmela Santarpia, Chiara Cullotta, Mariarosaria Galeano, Francesco Borgia, Federica Li Pomi, Vincenzo Arcoraci, Maria Lentini, Mariausilia Franchina, Mario Vaccaro, Giovanni Pallio, Natasha Irrera

**Affiliations:** 1 Department of Biomedical and Dental Sciences and Morphological and Functional Imaging, University of Messina, Via C. Valeria, 98125 Messina, Italy; federico.vaccaro92@gmail.com (F.V.); chiara.cullotta@studenti.unime.it (C.C.); mariarosaria.galeano@unime.it (M.G.); 2 Department of Medicine and Surgery, University of Enna “Kore”, Contrada Santa Panasia, 94100 Enna, Italy; federica.mannino@unikore.it; 3 Department of Human Pathology of Adult and Childhood Gaetano Barresi, University of Messina, Via C. Valeria, 98125 Messina, Italy; mariacarmela.santarpia@unime.it (M.S.); maria.lentini@unime.it (M.L.); mariausilia.franchina@studenti.unime.it (M.F.); 4 Department of Clinical and Experimental Medicine, University of Messina, Via C. Valeria, 98125 Messina, Italy; francesco.borgia@unime.it (F.B.); federicalipomi@hotmail.it (F.L.P.); vincenzo.arcoraci@unime.it (V.A.); mario.vaccaro@unime.it (M.V.); nirrera@unime.it (N.I.)

**Keywords:** melanoma, BRAF, genistein, curcumin

## Abstract

**Background/objectives**: Melanoma is one of the deadliest forms of malignant cancers; ultraviolet radiation exposure together with genetic mutations, such as BRAF, represent the main risk factors and are involved in metastatic dissemination. Previous studies demonstrated the anti-emetic and anti-proliferative effects of the flavonoid genistein and the turmeric curcumin in cancers. This study aimed at investigating the anticancer effects of curcumin, genistein and their association in melanoma cells. **Methods:** Human A375 and CHL-1 cell lines were cultured and treated with different concentrations of curcumin or genistein or curcumin + genistein for 24 h according to IC50. **Results**: Genistein and curcumin induced cell death, as demonstrated by MTT assay and FDA/PI staining. The anti-apoptotic protein Bcl-2 was significantly reduced after curcumin and curcumin + genistein treatment, but unexpectedly not with genistein alone. Curcumin and genistein significantly increased DNA fragmentation, thus indicating apoptosis induction. Moreover, comet assay confirmed that curcumin and genistein stimulated cell death, as quantified by measuring the displacement between the ‘comet head’ and the resulting ‘tail’. FAK protein expression was significantly reduced by genistein and curcumin in CHL-1 cells and after the treatment with genistein + curcumin in the most aggressive A375 cells. These anti-proliferative effects were confirmed by scratch assay and phospho-p38 reduction. Moreover, both curcumin and genistein alone and in association inhibited cell adhesion, thus indicating that these nutraceuticals could reduce invasion and metastasis. **Conclusion**: The obtained results provided new insights for the anticancer effects of genistein and curcumin, which could be used to improve therapeutic adherence and drug response.

## 1. Introduction

**Figure 1:**
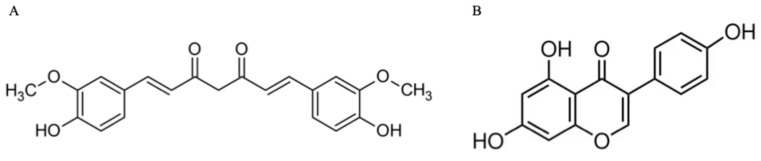
Chemical structures of curcumin (**A**) and genistein (**B**).

Melanoma is one of the deadliest forms of malignant skin cancers arising from melanocytes, responsible for a high rate of metastasis also as a consequence of the lack of no life-saving treatment for patients with advanced melanoma. In fact, the five-year survival rate exceeds 95% in the early stages of the disease, but survival rarely exceeds one year in the later stages [1]. The incidence of melanoma has considerably increased over the years, with a prevalence of approximately 300,000 cases per year and an estimate of approximately 470,000 new expected cases in 2040, with over 60,000 deaths each year [2]. Ultraviolet radiation exposure represents the main risk factor because of the stimulation of melanin production but also genetic mutations may play a significant role in the onset of melanoma as well as in the metastatic dissemination [3,4]. The most represented mutations depend on hereditary or sporadic origin, although some mutations such as that involving cyclin-dependent kinase inhibitor 2A (CDKN2A), a gene that encodes a 156 amino acid, 16 kD cell-cycle inhibitor protein, is common in familial and sporadic melanomas. The mutant p16CDKN2A is incapable of establishing stable complexes with CDK4/6 enzyme, consequently leading to an inadequate inhibition of cell progression during improper mitotic divisions. In addition, TP53, telomerase reverse transcriptase (TERT), BRAF and NRAS are common mutations observed in melanoma as well as in other cell types [5,6,7,8]. In particular, NRAS gene mutations occur in 20–30% of melanomas and rarely coexist with BRAF V600E, appearing together in 1% of cases only. This mutation, commonly found on exon 1 (codon 12) and exon 2 (codon 61), activates the MAP kinase pathway, thus causing the activation of the activated GPT state of the receptor tyrosine kinase. However, the largest molecular subtype is that defined by the presence of BRAF mutations, observed in approximately 50% of cutaneous melanomas. The most frequent BRAF mutation is V600E (approximately 85% of cases), followed by V600K (approximately 10%) and V600R (2%), which is anti-correlated with NRAS mutations. BRAF mutations may be also due to UV exposure and an association between BRAF mutation and clinical-pathological features has been detected, as well as an inverse relationship between BRAF mutation and age [9]. In the last 10 years, the treatment of melanomas improved with the adoption of monoclonal antibodies use, such as Ipilimumab and Pembrolizumab (both PD-1 inhibitors), BRAF inhibitors Dabrafenib and Vemurafenib and Trametinib (MEK inhibitor) [10]. These drugs act through checkpoint inhibition and targeted proliferation arrest in melanomas with BRAF mutation. The long-term treatment with BRAF and MEK inhibitors in association achieves a 5-year overall survival (OS) of 34% and a 5-year disease-free survival (PFS) of 19% [11]. However, these drugs not only are expensive but also fail to improve patient survival and their administration may cause adverse effects, such as immune-mediated colitis, hepatotoxicity and nephritis. The discovery of new therapies would be useful to guarantee better results and a higher disease-free survival rate. In this context, a growing interest was addressed to the use of phytochemicals and/or biologically active compounds of natural origin, thanks to their anti-emetic, antioxidant and anti-proliferative effects [12]. Previous experimental data showed that the phytochemicals flavonoids inhibit melanoma cells growth through the modulation of PI3K/AKT/mTOR and Wnt/β-catenin pathways [13,14]. Moreover, curcumin, a compound found in turmeric, showed promising results in the melanoma field, thus inhibiting cell growth and the metastatic potential by modulating the NF-κB pathway and microRNAs [15,16]. Despite the promising evidences obtained from preclinical studies, the clinical translation of phytochemicals use for the treatment of melanoma is still in early stages. Further research is needed to optimize the use of phytochemicals as complementary or alternative therapies for melanoma and to better understand the mechanism of action of these natural compounds, also in different conditions; therefore, the aim of this study was to investigate the anti-proliferative effects of the phenolic curcumin and genistein (Figure 1) in melanoma cell lines.

## 2. Materials and Methods

### 2.1. Cell Cultures

Human A375 CRL-1619 (a cell line exhibiting epithelial morphology that was isolated from the skin of a 54-year-old, female patient with malignant melanoma) and CHL-1 CRL-9446 (primary melanoma cells, homozygous c. 578A > G protein sequence p. H193R) cell lines were acquired by ATCC (ATCC Manassas, Manassas, VA, USA). Dulbecco’s Modified Eagle’s Medium (Sigma-Aldrich, St. Louis, MO, USA) with 1% of antibiotic (penicillin/streptomycin, Sigma-Aldrich, St. Louis, MO, USA), 10% fetal bovine serum (FBS) (ATCC Manassas, Manassas, VA, USA) was used for cells cultures, which were incubated at 37 °C with 5% CO_2_. The culture medium was replaced every 2 days and each time confluence was reached, cells were re-plated.

### 2.2. MTT Assay

MTT assay (3-(4,5-dimethylthiazol-2-yl)-2,5-diphenyltetrazolium Bromide) was used for cell viability evaluation; in detail, A375 and CHL-1 cells were seeded at the density of 1 × 10^5^ cells/well in a 96-well plate; upon reaching confluence, A375 were treated with curcumin (5, 10, 20, 40, 80 μM), genistein (12.5, 25, 50, 100, 200 μM) and curcumin + genistein (20 + 50, 40 + 50, 20 + 100, 40 + 100 μM) for 24 h. CHL-1 cells were treated with curcumin (6.25, 12.5, 25, 50 μM), genistein (12.5, 25, 50, 100 μM) and curcumin + genistein (12.5 + 50, 12.5 + 100, 25 + 50, 25 + 100, 25 + 200 μM) for 24 h.

Tetrazolium dye MTT 3-(4,5-dimethylthiazol-2-yl)-2,5-diphenyltetrazolium bromide (5 mg/mL) (Sigma Aldrich, St. Louis, MO, USA) was dissolved in sterile PBS and 20 μl of this solution was added to each well five hours before the end of the treatment; formazan crystals were suspended with 200 µL of dimethyl sulfoxide (DMSO). Cell viability was detected at λ 540 and 620 nm by using the VICTOR Multilabel Plate Reader (Perkin Elmer, Waltham, MA, USA) m. Results are expressed as the percentage of cell viability compared to untreated cells [17].

### 2.3. Cell Treatments

A375 and CHL-1 cells were plated (1 × 10^6^ cells/well) in 6-well plates and incubated at 37 °C with a percentage of 5% CO_2_ overnight. The day after, both A375 and CHL-1 cells were treated with IC50 doses of curcumin (40 or 25 μM), genistein (50 or 100 μM), and curcumin + genistein (40 + 100 or 25 + 50 μM), obtained from the MTT assay for 24 h. Cells were then collected for further molecular analyses.

### 2.4. PI Staining

Both cell lines were plated (5 × 10^5^ cells/well) in 24-well plates; A375 cells were treated with curcumin (40 µM), genistein (50 μM) and curcumin + genistein (40 + 100 μM), whereas CHL-1 cells were treated with curcumin (25 µM), genistein (100 μM) and curcumin + genistein (25 + 50 μM) for 24 h. Following washing with sterile PBS, PI staining solution (2 mg/mL) was used for 5 min at room temperature in the dark to stain cells. Dead cells were observed with a fluorescence microscope and ImageJ 1.53e software for Windows (Softonic, Barcelona, Spain) was used to calculate positive cells number [18]

### 2.5. DNA Laddering Assay

Both cell lines were seeded (1 × 10^6^ cells/well) in 6-well plates; A375 cells were treated with curcumin (40 µM), genistein (50 μM) and curcumin + genistein (40 + 100 μM), whereas CHL-1 cells were treated with curcumin (25 µM), genistein (100 μM) and curcumin + genistein (25 + 50 μM) for 24 h. DNA was extracted following the manufacturer’s instructions and DNA fragments were electrophoresed in 1% agarose gel at 80 V for 30 min. The gel was visualized under UV light by using a Bio-Rad UV transilluminator 2000 (Hercules, CA, USA) and images were captured with 13 mm ultra wide-angle camera (8 MP resolution, 2160 × 3840 pixels, ISO 40, f/2.2, 1/45 s shutter speed). The extent of DNA damage was evaluated by assessing the pattern of DNA fragmentation. The presence of a ladder-like pattern indicated internucleosomal cleavage typical of apoptotic cells, whereas a smear pattern was associated with random DNA degradation. The degree of DNA damage was qualitatively compared between treated and control samples based on band intensity and distribution.

### 2.6. Western Blot

At the end of the treatment period, A375 and CHL-1 cells were collected with RIPA buffer (25 mM Tris/HCl, pH 7.4; 1.0 mM EGTA; 1.0 mM EDTA) containing NP40 (1%), phenyl methylsulfonyl fluoride (PMSF, 0.5%), aprotinin, leupeptin and pepstatin (10 μg/mL each) and centrifuged at 15.000 rpm for 15 min at 4 °C. Supernatants were used to measure the total protein content by using the Bio-Rad protein-assay kit (BioRad, Hercules, CA, USA). Proteins (30 μg) were loaded and run by electrophoresis in a 10% sodium dodecyl sulphate (SDS) polyacrylamide gel to be then transferred on PVDF membranes (Amersham, Little Chalfont, UK) with a specific Transfer Buffer at 200 mA for 1 h. Following washing with TBS 0.1% and blocking with 5% non-fat dry milk, membranes were incubated with Bcl-2 (Abcam, Cambridge, UK), Bax (BioVision, Milpitas, CA, USA), phospho-Fak (Cell signaling Danvers, MA, USA), phospho-JNK (Cell signaling Danvers, MA, USA) and phospho-p38 (Cell signaling Danvers, MA, USA), antibodies diluted in TBS-0.1% Tween overnight. The day after, a secondary goat anti-rabbit antibody (GeneTex, Irvine, CA, USA) conjugated with peroxidases was added for 1 h, after washing with TBS-0.15% Tween buffer. Washed membranes were analyzed by the enhanced chemiluminescence system (LumiGlo reserve, Seracare, Milford, MA, USA) and the protein signal was quantified by scanning densitometry system (C-DiGit, Li-cor, Lincoln, NE, USA). The results were expressed as integrated intensity and β-actin (Cell Signaling, Danvers, MA, USA) was used as control of the equal loading [19,20,21,22,23].

### 2.7. Cell Death Evaluation by Comet Assay

DNA damage was evaluated with a comet assay kit (Abcam, Cambridge, UK). Following treatment, cells were collected and centrifuged at 700× g for 2 min; cell pellets were washed with ice-cold sterile PBS and re-suspended at 1 × 10^5^ cells/mL in ice-cold sterile PBS. Cells were combined with Comet Agarose at 1/10 ratio (*v*/*v*), 75 μL/well were transferred onto the top of the Comet Agarose Base Layer and kept at 4 °C in the dark for 15 min. Slides were transferred in a Lysis Buffer for 30 min at 4 °C in the dark followed by Alkaline Solution for 30 min at 4 °C in the dark. Slides were washed twice in TBE Electrophoresis Solution for 5 min and transferred to a horizontal electrophoresis chamber. After washing with 70% ethanol for 5 min, 100 μL/well of diluted Vista Green DNA Dye were added on the slides for 15 min. The slides were observed and photographed with a fluorescent microscope by using a FITC filter. The DNA damage is quantified by measuring the displacement between the genetic material of the nucleus (comet head) and the resulting tail by using ImageJ 1.53e software for Windows (Softonic, Barcelona, Spain). Tail DNA% = 100 × tail DNA intensity; Tail Olive Moment = tail DNA% × length of tail. Tail moment length is measured from the center of the head to the center of the tail.

### 2.8. Intracellular ROS Levels Measurement

5-(and-6)-chloromethyl-2,7′-dichlorodihydrofluorescein diacetate (CM-H2DCFDA) probe was used to evaluate the accumulation of intracellular ROS in A375 and CHL-1 cells. CM-H2DCFDA probe 5 mM (Thermo Fisher, Carlsbad, CA, USA) was added into each well for 1 h at 37 °C; cells were washed 2–3 times with sterile PBS and observed with a fluorescent microscope. Fluorescence quantification was performed by using ImageJ 1.53e software for Windows (Softonic, Barcelona, Spain)

### 2.9. Adhesion Assay

Cells were plated (5 × 10^5^ cells/well) in 12-well plates for 12 h to be treated with curcumin, genistein and curcumin + genistein for 24 h. Following harvesting, cells were re-seeded in 24-well plates for 3 h. Unattached cells were firstly removed; PBS was used for washing attached cells and cells were fixed with 4% paraformaldehyde at room temperature for 15 min. Cristal violet staining solution (Beyotime, Haimen, Jiangsu, China) was used for 10 min at room temperature. PBS was again used for washing and the OD value was measured at 570 nm by using a microplate reader (C-DiGit, Li-cor, Lincoln, NE, USA). The value of adhered cells was calculated in percentage with respect to controls [24].

### 2.10. Wound Healing Analysis

A375 and CHL-1 cells were seeded (1 × 10^6^ cells/well) in 6-well plates; following 24 h of adherence, the medium was removed, and cell monolayers were scratched by manually using a sterile P1000 micropipette tip. Cells were washed three times with sterile PBS, cultured in a serum-free medium and then treated with genistein, curcumin and genistein + curcumin for 24 h. Cell migration was observed and photographed at the time of scratch and following 24 h by using the Olympus EP50 microscope (Life Science, Amstelveen, The Netherlands) and the wound healing rate was calculated by using ImageJ 1.53e software for Windows (Softonic, Barcelona, Spain) and expressed as % Wound Closure = [(Initial Wound Area-Wound Area at Time T1)/Initial Wound Area] * 100 [15].

### 2.11. Immunofluorescence

A375 cells were seeded onto a glass coverslip and fixed in 4% of paraformaldehyde (PFA) in 0.2 M phosphate buffer (pH 7.4) for 2 h at RT. After rinsing, cells were preincubated with 0.3% triton X-100 in PBS for 10 min to permeabilize the membranes and with 1% bovine serum albumin (BSA) in PBS (30 min at RT) for blocking nonspecific binding sites. Cells were incubated overnight at 4 °C with rabbit polyclonal MMP9 (1:250 dilution Invitrogen, Waltham, MA, USA) and cyclin D1 antibodies (1:400 dilution) (Cell signaling Danvers, MA, USA). After removing the excess of primary antibody with PBS, a FITC-conjugated IgG anti-rabbit antibody was used (Jackson ImmunoReseach Laboratories, Inc., West Grove, PA, USA) for 1 h at RT. DAPI (Sigma Aldrich, St. Louis, MO, USA) was used with 1:1000 dilution for 10 min at RT to stain nuclei. Cells were then washed in PBS, and the coverslips were mounted on slides. Images were observed and acquired by using Zeiss LSM 5 Duo confocal laser scanning microscope (Carl Zeiss, Iena, Germany) and a resolution of 8 bits into an array of 2048 × 2048 pixels was applied. Optical sections were obtained with an Argon laser (wave-length = 488 nm) at a 762 s scanning speed with up to 8 averages. Contrast and brightness were established by evaluating the most brightly labeled pixels and choosing the settings that allowed clear visualization of the structural details while keeping the pixel intensity at its highest (200). The parameters were only selected on the scans made on controls (untreated cells) and the same parameters were then maintained for other samples. Each image was acquired within 62 s to minimize the photodegradation. Digital images were cropped and figures were prepared by using Adobe Photoshop 7.0 (Adobe System, Palo Alto, CA, USA)

### 2.12. Statistical Analysis

All the results are expressed as means ± standard deviation (SD). The experiments were replicated thrice, and all assays were performed in duplicate to ensure reproducibility. The differences between groups were evaluated by applying the one-way ANOVA test and Tukey’s post-test. A *p* value less than 0.05 was considered significant. Graphs were prepared with GraphPad Prism Version 8.0 for macOS (GraphPad Software Inc., La Jolla, CA, USA).

## 3. Results

### 3.1. Curcumin and Genistein Reduce Melanoma Cell Viability

**Figure 2:**
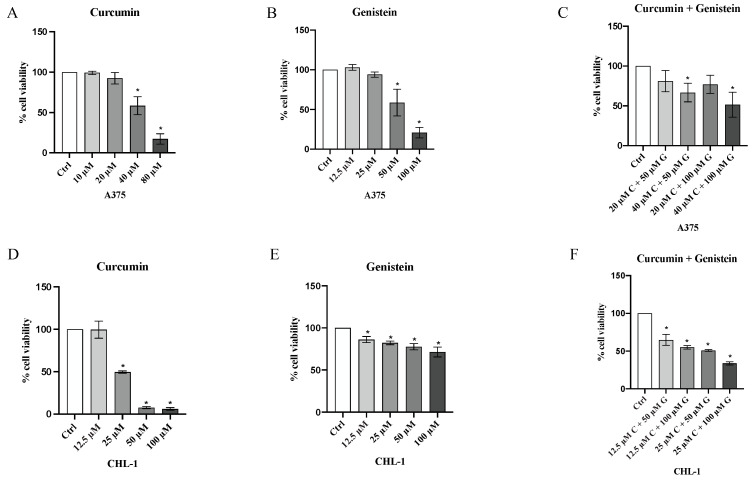
Cytotoxicity evaluated by MTT assay in A375 cell line treated with curcumin (**A**) (10, 20, 40 and 80 μM), genistein (**B**) (12.5, 25, 50 and 100 μM) and curcumin + genistein (**C**) (20 + 50, 40 + 50, 20 + 100, 40 + 100 μM) and in CHL-1 cell line treated with curcumin (**D**) (12.5, 25, 50 and 100 μM), genistein (**E**) (12.5, 25, 50 and 100 μM) and curcumin + genistein (**F**) (12.5 + 50, 12.5 + 100, 25 + 50 and 25 + 100 μM) for 24 h. The data are expressed as means ± SD; * *p* < 0.05 vs. Ctrl.

**Figure 3:**
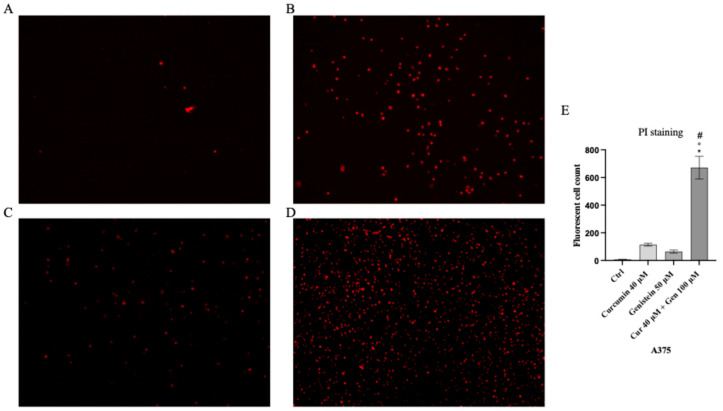
The figures show cell viability evaluated by PI staining of A375 untreated (**A**), and treated with curcumin (**B**), genistein (**C**) and curcumin + genistein (**D**). In panels (**A**–**D**), the red staining indicates dead cells. Panel (**E**) shows A375 fluorescent cell count. All images were captured at 10× of magnification. The data are expressed as means ± SD. * *p* < 0.05 vs. CTRL; # *p* < 0.05 vs. curcumin; ° *p* < 0.05 vs. genistein.

**Figure 4:**
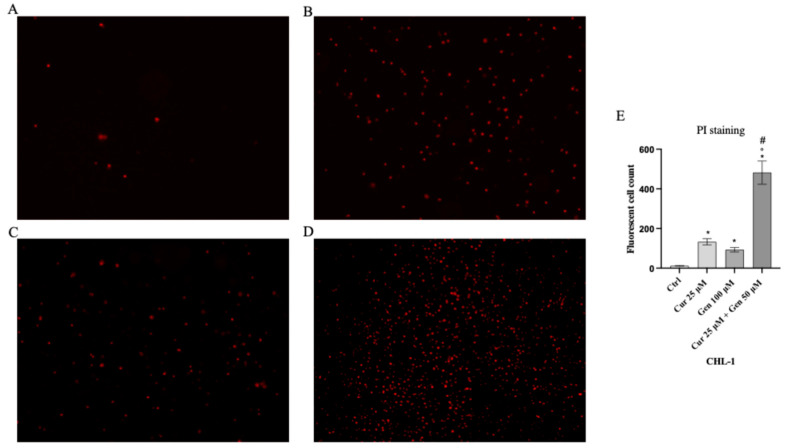
The figures show cell viability evaluated by PI staining of CHL-1 untreated (**A**), and treated with curcumin (**B**), genistein (**C**) and curcumin + genistein (**D**). In panels (**A**–**D**), the red staining indicates dead cells. Panel (**E**) shows CHL-1 fluorescent cell count. All images were captured at 10× of magnification. The data are expressed as means ± SD. * *p* < 0.05 vs. CTRL; # *p* < 0.05 vs. curcumin; ° *p* < 0.05 vs. genistein.

Cell viability was calculated as an indirect assessment based on metabolic activity through MTT assay in both cell lines following treatment with curcumin and genistein alone or in combination at different concentrations. The viability of A375 cells was significantly reduced following treatment with curcumin (40 and 80 μM; Figure 2A). As shown in Figure 2B, genistein (50 and 100 μM) markedly reduced cell viability of A375 cells. Moreover, the combination of the tested compounds affected A375 cell viability at the concentrations of 40 + 50 and 40 + 100 μM (Figure 2C). Curcumin significantly reduced CHL-1 cell viability at concentrations of 25 and 50 μM (Figure 2D); instead, cell viability was markedly reduced at all tested concentrations of genistein (Figure 2E). Furthermore, the combination of curcumin + genistein affected cell viability starting from 12 + 50 μM (Figure 2F). PI staining was performed to confirm MTT results: A375 cells treated with curcumin (40 μM), genistein (50 μM) and in particular with their combination (40 and 100 μM) for 24 h showed many nuclei stained with PI (Figure 3A–D). Stained nuclei were also observed in CHL-1 cell line, although at different concentrations, 25, 100 and 25 + 50 μM (Figure 4A–D). The graphs reported in Figure 3E and Figure 4E represent A375 and CHL-1 PI positive cell count.

### 3.2. Curcumin, Genistein and Their Combination Induce Cell Cancer Death

**Figure 5:**
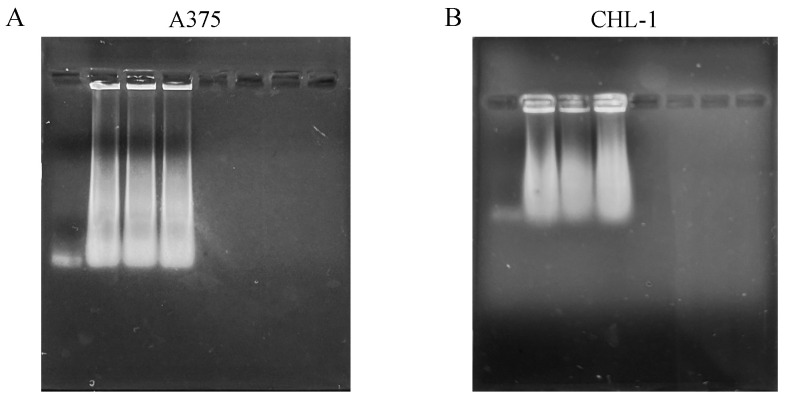
DNA fragmentation of melanoma cells. The panels show DNA fragmentation in A375 (**A**) and CHL-1 (**B**) cells untreated and treated with curcumin, genistein and curcumin + genistein.

DNA fragmentation represents one of the hallmarks of apoptosis and was evaluated with DNA electrophoresis. Both curcumin and genistein alone and in combination significantly increased DNA fragmentation in both cell lines, as shown in Figure 5.

**Figure 6:**
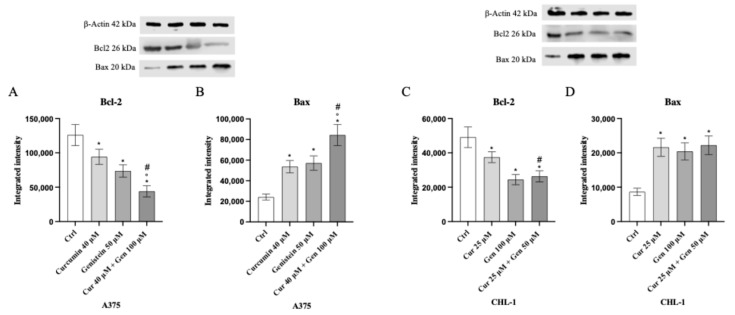
The graphs show Bcl-2 (**A**) and Bax (**B**) protein expression in A375 cells and Bcl-2 (**C**) and Bax (**D**) in CHL-1 cells treated with curcumin, genistein and curcumin + genistein. The data are expressed as means ± SD. * *p* < 0.05 vs. CTRL; # *p* < 0.05 vs. curcumin; ° *p* < 0.05 vs. genistein.

In addition, in order to confirm apoptosis induction, Bcl-2 and Bax expression was evaluated by Western blot analysis. The anti-apoptotic marker Bcl-2 was significantly following treatment with curcumin, genistein and in particular with curcumin + genistein in both A375 and CHL-1 cells compared to untreated cells. Curcumin, genistein and curcumin + genistein significantly stimulated Bax expression in A375 and CHL-1 cells compared to untreated cells (Figure 6).

**Figure 7:**
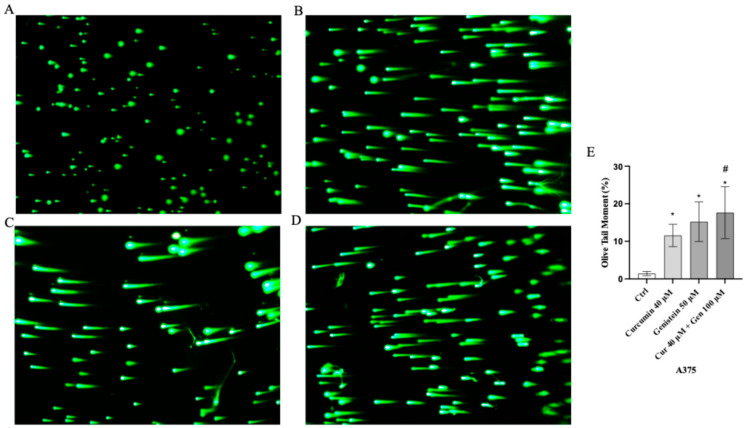
Cellular DNA damage measured by comet assay from Ctrl (**A**), curcumin (**B**), genistein (**C**) and curcumin + genistein (**D**) in A375 cells. All images were captured at 10× of magnification. The graph (**E**) shows the DNA damage represented as Tail Olive Moment. The data are expressed as means ± SD. * *p* < 0.05 vs. Ctrl; # *p* < 0.05 vs. curcumin.

**Figure 8:**
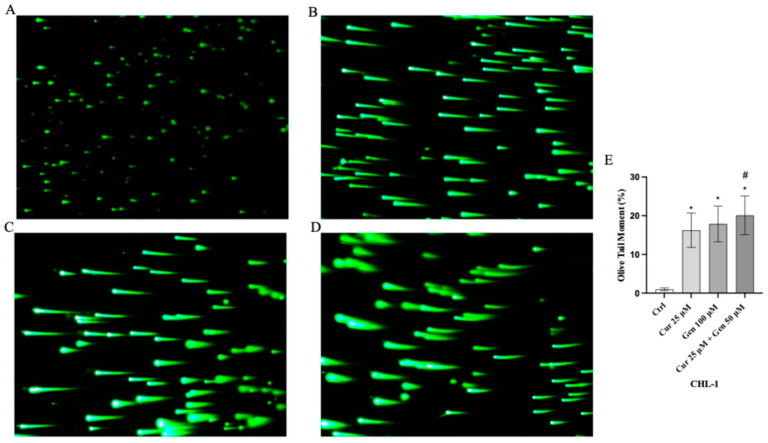
Cellular DNA damage measured by comet assay from Ctrl (**A**), curcumin (**B**), genistein (**C**) and curcumin + genistein (**D**) in CHL-1 cells. All images were captured at 10× of magnification. The graph (**E**) shows the DNA damage represented as Tail Olive Moment. The data are expressed as means ± SD. * *p* < 0.05 vs. Ctrl; # *p* < 0.05 vs. curcumin.

Moreover, comet assay confirmed that curcumin and genistein alone or in combination significantly increased cell death in both cell lines, as quantified by measuring the displacement between the genetic material of the nucleus (‘comet head’) and the resulting ‘tail’ (Figure 7A–D and Figure 8A–D). In fact, the analysis of Tail Olive Moment in CHL-1 and A375 cells indicated an increasing median value, as shown in Figure 7E and Figure 8E.

### 3.3. Curcumin, Genistein and Their Combination Increase Oxidative Stress

**Figure 9:**
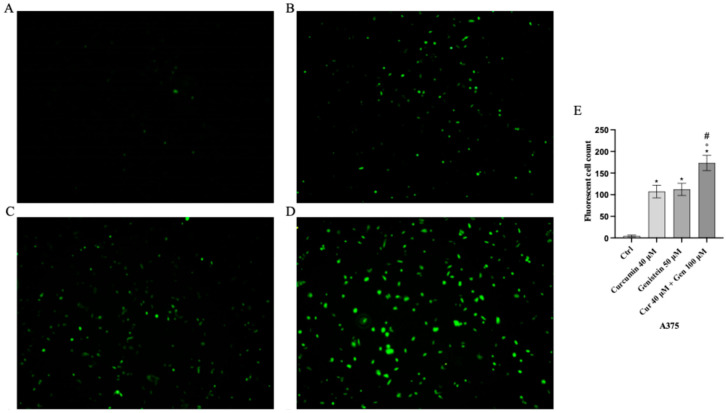
Intracellular ROS accumulation evaluated by CM-H2DCFDA fluorescent probe from Ctrl (**A**), curcumin (**B**), genistein (**C**) and curcumin + genistein (**D**) in A375 cells. All images were captured at 10× of magnification. Panel (**E**) Shows the number of fluorescent cells. The data are expressed as means ± SD. * *p* < 0.05 vs. CTRL; # *p* < 0.05 vs. curcumin; ° *p* < 0.05 vs. genistein.

**Figure 10:**
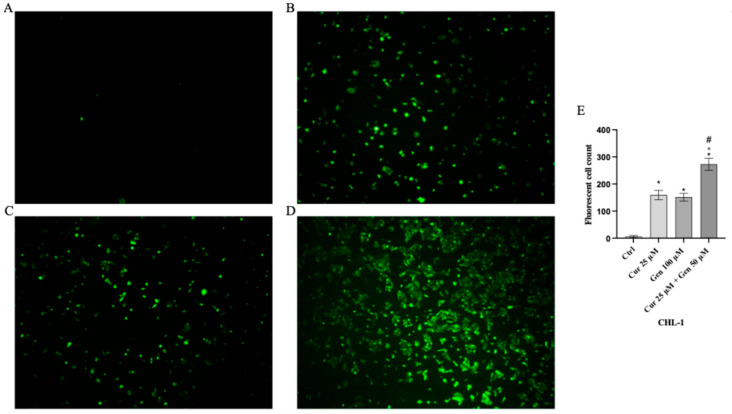
Intracellular ROS accumulation evaluated by CM-H2DCFDA fluorescent probe from CTRL (**A**), curcumin (**B**), genistein (**C**) and curcumin + genistein (**D**) in CHL-1 cells. All images were captured at 10× of magnification. Panel (**E**) Shows the number of Fluorescent cells. The data are expressed as means ± SD. * *p* < 0.05 vs. CTRL; # *p* < 0.05 vs. curcumin; ° *p* < 0.05 vs. genistein.

Intracellular ROS production was significantly increased in A375 and CHL-1 cells treated with curcumin and genistein compared to untreated cells; the coincubation of curcumin and genistein showed a greater effect than that observed when the tested compounds were used alone, as demonstrated by the increase in fluorescent signal showed in Figure 9 and Figure 10.

### 3.4. Curcumin and Genistein Influence Melanoma Cell Adhesion

**Figure 11:**
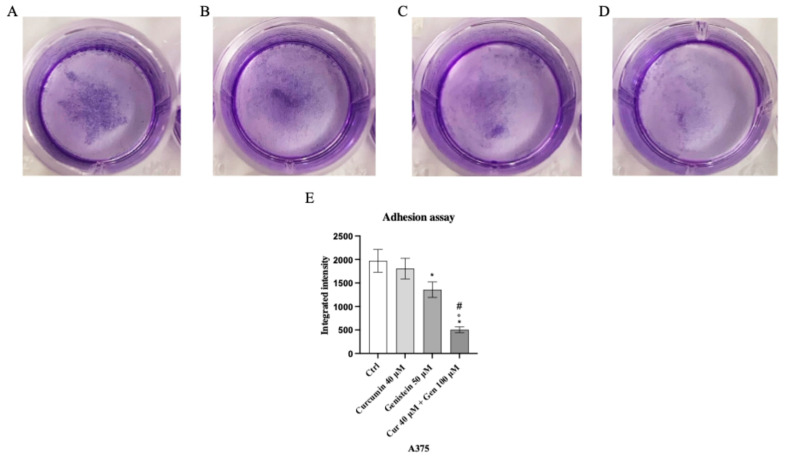
The images show the A375 attached cells untreated (**A**) and treated with curcumin (**B**), genistein (**C**) and curcumin + genistein (**D**) and stained with crystal violet. Panel (**E**) shows the OD value measured at 570 nm using a microplate reader. The data are expressed as means ± SD. * *p* < 0.05 vs. Ctrl; # *p* < 0.05 vs. curcumin; ° *p* < 0.05 vs. genistein.

**Figure 12:**
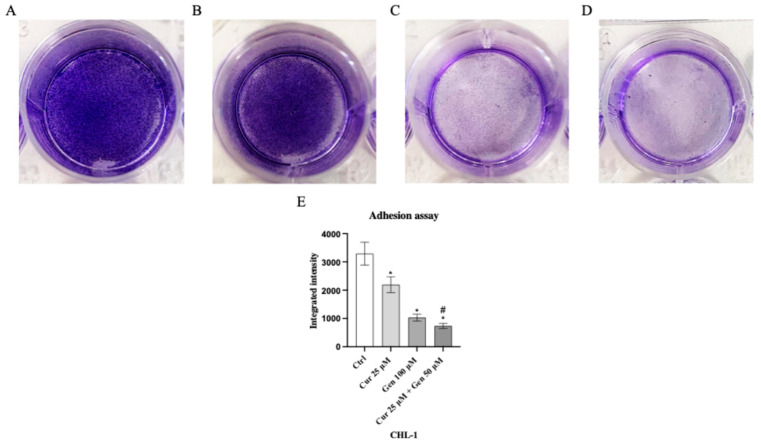
The images show the CHL-1 attached cells untreated (**A**) and treated with curcumin (**B**), genistein (**C**) and curcumin + genistein (**D**) and stained with crystal violet. Panel (**E**) shows the OD value measured at 570 nm using a microplate reader. The data are expressed as means ± SD. * *p* < 0.05 vs. Ctrl; # *p* < 0.05 vs. curcumin.

Genistein at a concentration of 50 μM and in particular the combination of curcumin + genistein (40 + 100 μM) reduced cell adhesion, showing a rate inhibition of 25% and 75% in A375 cell line compared to the untreated cells (Figure 11). Moreover, cell adhesion was significantly reduced also in CHL-1 cells treated with curcumin (25 μM), genistein (100 μM) and in particular with their combination, showing inhibition levels of 33%, 66% and 73%, approximatively, compared to untreated cells (Figure 12).

### 3.5. Curcumin, Genistein and Their Combination Reduce Cell Migration

**Figure 13:**
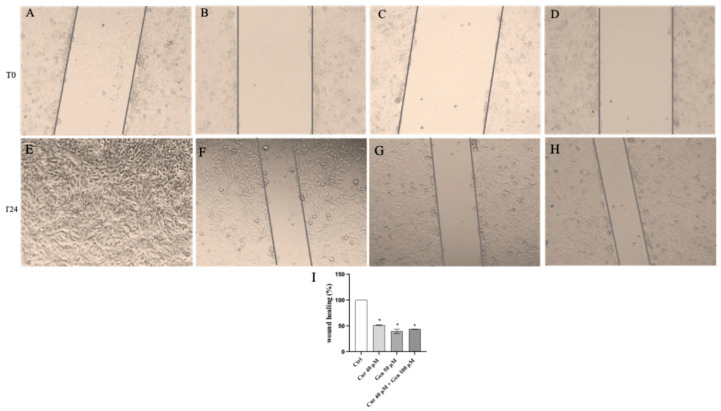
The panel shows Wound Healing Analysis in A375 cells at T0 (**A**–**D**) and following 24 h (**E**–**H**) in cells treated with curcumin (**F**), genistein (**G**) and curcumin + genistein (**H**) The graph in (**I**) summarizes the percentage of wound healing in all experimental groups. All images were captured at 10× of magnification. The data are expressed as means ± SD. * *p* < 0.05 vs. Ctrl.

Cell mobility was observed at time 0 and following 24 h of curcumin and genistein treatment and after their use in combination: this result was observed in both cell lines. A continuous mobility was detected in the untreated A375 cells (Figure 13E); instead, cell migration was markedly reduced following treatment with curcumin, genistein or their combination in A375 cells (Figure 13F–I).

**Figure 14:**
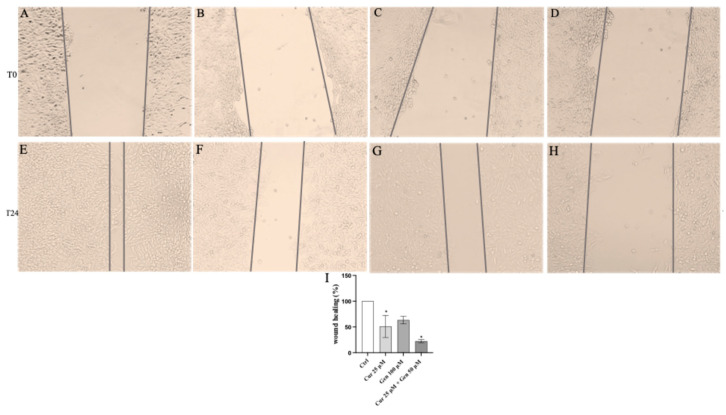
The panel shows Wound Healing Analysis in CHL-1 cells at T0 (**A**–**D**) and following 24 h (**E**–**H**) in cells treated with curcumin (**F**), genistein (**G**) and curcumin + genistein (**H**) The graph in (**I**) summarizes the percentage of wound healing in all experimental groups. All images were captured at 10× of magnification. The data are expressed as means ± SD. * *p* < 0.05 vs. Ctrl.

In addition, continuous mobility of CHL-1 cells was observed in the untreated cells following 24 h (Figure 14E) whereas, curcumin, genistein, and in particular their combination markedly reduced cell migration compared to the control group (Figure 14F–I).

**Figure 15:**
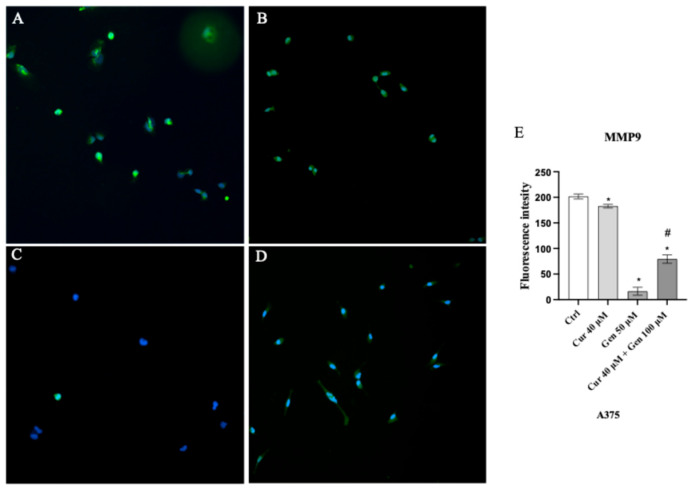
Immunofluorescence images of MMP9 (green fluorescence) for Ctrl (**A**), curcumin (**B**), genistein (**C**) and curcumin + genistein (**D**) treated A375 cells. Panel (**E**) shows fluorescent intensity observed in ten cells. The data are expressed as means ± SD. * *p* < 0.05 vs. Ctrl; # *p* < 0.05 vs. curcumin.

An intense fluorescence pattern was observed in control cells (Figure 15A); cells treated with curcumin and in particular with genistein showed a significant decrease in the fluorescence intensity (Figure 15B,C). Also, the concomitant use of curcumin and genistein significantly reduced MMP9 expression as showed in Figure 15D.

**Figure 16:**
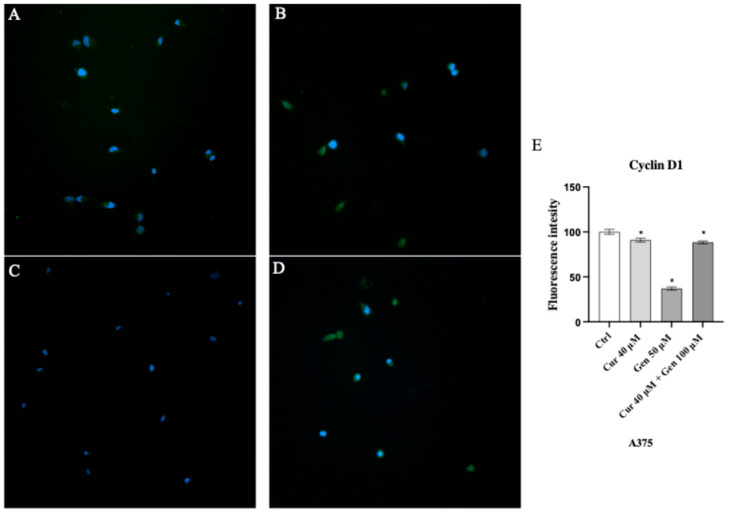
Immunofluorescence images of cyclin D1 (green fluorescence) for Ctrl (**A**), curcumin (**B**), genistein (**C**) and curcumin + genistein (**D**) treated A375 cells. Panel (**E**) shows fluorescent intensity observed in ten cells. The data are expressed as means ± SD. * *p* < 0.05 vs. Ctrl.

In order to investigate the effects of curcumin and genistein on cell cycle we also evaluated the protein expression of cyclin D1. A slight reduction was observed following curcumin treatment (Figure 16B), whereas a strong reduction in fluorescent intensity was observed in genistein treated cells (Figure 16C).

**Figure 17:**
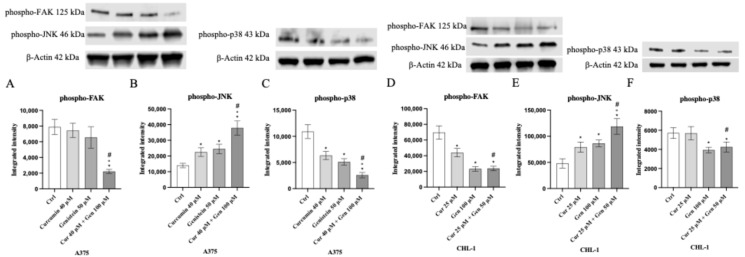
The graphs show phospho-FAK, phospho-JNK and phospho-p38 protein expression evaluated by Western blot analysis in A375 (**A**–**C**) and CHL-1 (**D**–**F**) cells. The data are expressed as means ± SD. * *p* < 0.05 vs. Ctrl; # *p* < 0.05 vs. curcumin; ° *p* < 0.05 vs. genistein.

MAPKs and FAK cell signaling were studied as pathways involved in cell proliferation, tumor migration, invasion and metastasis. FAK protein expression was significantly reduced following curcumin + genistein treatment compared to untreated cells in A375 cell line (Figure 17A). All tested compounds and in particular their combination were able to significantly reduce phospho-p38 expression and increase phospho-JNK protein levels compared to untreated cells (Figure 17B–C). Moreover, FAK and phospho-p38 levels were markedly reduced following genistein and curcumin + genistein treatment, whereas all tested compounds were able to significant increase phospho-JNK expression compared to untreated cells in CHL-1 cell line (Figure 17D–F).

## 4. Discussion

Curcumin and genistein may play different biological functions and several effects have been demonstrated to date, including the modulation of cell proliferation particularly active in cancers, thus promoting invasion and metastasis formation [25,26,27]. Melanoma is a particularly aggressive tumor with a poor prognosis that may be influenced by genetic factors (i.e., BRAF mutation) [28], in fact BRAF wild-type (CHL-1) and mut (A375) cells have been widely used in the field of melanoma [29,30,31,32] and these cell lines were also used in our experimental model to provide a translation impact. Curcumin and genistein were already used in other cell lines including normal human melanocytes. In this context the use of curcumin induced nonspecific toxic effects and melanogenesis inhibition, with the involvement of MITF, ERK, PI3K/Akt and GSK 3β pathways [33]. This result was also confirmed by an in vivo study on Zebrafish embryos that showed curcumin anti-melanogenic properties and dose-dependent toxic effects during early development [34]. Also genistein may play both melanogenic and anti-melanogenic effects in normal versus transformed melanocytes through modulation of MITF transcription and tyrosinase-related proteins such as TRP-1 and DCT/TYR-related protein-2 [35]. These evidences, as well as the results obtained from this study allow us to hypothesize that the outcome depends on cell type and context. In this experimental setting, curcumin and genistein were used alone and for the first time in association to evaluate their anticancer effects; cell viability was studied by MTT and PI assays, which demonstrated that these natural products induced cell death in both A375 and CHL-1 cells, respectively, in accordance with previous experiments that already showed genistein and curcumin effects on cancer cells [36,37,38,39,40,41]. The promotion of cell death processes may consequently inhibit tumor proliferation and invasiveness that represent two of the main features of the aggressive melanoma. The anti-inflammatory and antioxidant effects of genistein and/or curcumin are well known, as well as their ability in modulating apoptosis, as cell death mechanism [42,43,44,45,46,47]. In this experimental setting, we demonstrated that both genistein and curcumin alone and in combination induced cell cancer death compared to untreated cells in both cell lines, and, in particular, this effect increased when genistein and curcumin were used together. In fact, the anti-apoptotic protein Bcl-2 was significantly reduced after treatment with curcumin or with curcumin + genistein, thus confirming apoptosis induction in both A375 and CHL-1 cells. Apoptotic cell death is not only regulated by Bcl-2 protein family, but it is also characterized by a typical pattern of DNA fragmentation known as “apoptotic ladder”, resulting from inter-nucleosome cleavage of genomic DNA. In this context, Jiang et al. [48] demonstrated that curcumin can induce DNA double strand breaks by DNA fragmentation analysis and in accordance with these results we observed that curcumin and genistein, alone or in combination, induced DNA fragmentation in both A375 and CHL-1 malignant cells. Cell proliferation is regulated by cell cycle whose progression from the G1 to the S phase is mediated by cyclins, as cyclin D1, which is overexpressed in different tumors and also in melanoma [49]. A previous study conducted by Srivastava et al. showed the effects of curcumin in down-regulating cyclin D1, together with other pathways, thus resulting in the suppression of cell proliferation [50]. Genistein may induce G0/G1 cell arrest [51] as well as cell cycle arrest from G1 to S phase depending on the used concentration [52]. We found that curcumin, but in particular genistein as well as the association of curcumin and genistein significantly reduced cyclin D1 expression in A375 cell line. In addition, MMP9 expression was significantly decreased following curcumin and genistein administration alone and in association as already demonstrated in several papers that investigated their anti-proliferative effects both in vitro and in vivo [53,54] Generally, cancer cells frequently exhibit increased levels of ROS, which can have both tumor-promoting and tumor-suppressing effects depending on their concentration and the cellular environment [55]. ROS are also considered signaling molecules that support cancer cell growth, migration as well as resistance to therapy at low and moderate levels. In contrast, high levels of ROS can exceed cellular antioxidant capacity, thus causing oxidative stress and triggering cell death through mechanisms, such as apoptosis. To cope with elevated ROS, cancer cells often activate antioxidant defenses in order to balance their metabolic processes [56]. This dual nature of ROS represents a therapeutic opportunity, with treatment strategies focusing either on boosting ROS to induce cancer cell death or on interfering with ROS signaling to hinder tumor progression. By evaluating ROS accumulation with the CM-H2DCFDA fluorescent probe, we found that curcumin and genistein stimulated ROS release, in particular when used together thus let us to hypothesize that cell death was also induced by this compensatory mechanism. The mechanisms of cell death, proliferation and invasiveness are regulated by different intracellular cell signaling, such as that of the Focal Adhesion Kinase (FAK) and the p38 and JNK (c-Jun N-terminal kinase) MAP kinases [57,58]. Previous studies already demonstrated that genistein and curcumin may regulate these pathways [59,60], thus letting us to hypothesize that these natural products may also manage this invasive cancer through the modulation of these cell signaling. In agreement with the cited papers, our results demonstrated that FAK protein expression was significantly reduced following all treatments in BRAF wild-type cells, whereas this protein involved in the mechanisms of invasiveness and metastasis was significantly reduced following the treatment with the combination of genistein and curcumin in the most aggressive A375 cell line, only. All tested compounds and in particular their combination significantly decreased phospho-p38 expression compared to untreated cells in both cell lines although phospho-p38 expression was not modified by the administration of curcumin alone in CHL-1 cells. Both apoptosis and DNA damage may be induced by JNK [61,62] and, accordingly, we found that phospho-JNK expression was significantly increased following the treatment with curcumin and genistein alone and in particular with their association in both cell lines. Cell migration was also studied by scratch assay that showed cell migration inhibition following the treatment with genistein and curcumin: these results further confirm their anti-proliferative effects. The present data are in accordance with the evidences reported by Cui et al., that demonstrated the inhibitory effect of genistein at concentrations of 50 and 100 μM on cell mobility, although in a different cell line (B16F10 cells), and with the results described by Long Li et al., that showed that curcumin at concentrations of 50 μM and 100 μM significantly suppressed the migration of SK-MEL-1 cells following 24 h of treatment [19,63]. However, no data had previously demonstrated the inhibitory effect of genistein and curcumin in association in these cell lines. Moreover, both natural products alone and in association inhibited cell adhesion, thus indicating that genistein and curcumin may play an inhibitory effect on invasion and metastasis. On the other hand, a decrease in cell adhesion could also be a driver of increased metastatic capacity. In fact, by enabling detachment, reduced adhesion enables cancer cells to successfully seed new tumors. Consequently, restoring adhesion mechanisms or targeting adhesion pathways signaling may represent a promising therapeutic strategy to reduce metastasis and improve outcomes. Notably, adhesion strength could be considered a potential biophysical marker [64], but its correlation to cancer metastasis has only been demonstrated in vitro by using surrogates such as migration, velocity, and persistence. Further in vivo validation will be essential to confirm its use as a reliable biomarker in clinical setting.

## 5. Conclusions

The results obtained so far are promising and provide new insights for melanoma treatment by using nutraceuticals, at least in terms of adjuvant therapy or “medical foods”, also in advanced melanomas. The use of natural products could improve the therapeutic adherence as well as therapy effectiveness, thus avoiding relapses and reducing the already known side effects of traditional treatments [65,66,67,68]. However, further in vivo studies could help to characterize the mechanism of action of both genistein and curcumin in mutated BRAF cells and clarify their effects in order to hypothesize clinical trials for a future use in the clinical practice.

Despite the use of different methods to characterize genistein and curcumin effects, particularly in combination, this study shows some important limitations. Notably, the absence of computational studies and molecular docking analyses represents a significant gap in understanding the precise molecular interactions involved. Moreover, the lack of deeper investigation into specific markers related to invasiveness and cell cycle regulation limits the comprehensive understanding of the pathways modulated by these compounds. Addressing these limitations in future studies will be essential to elucidate the molecular mechanisms underlying the observed effects and to better define the therapeutic potential of genistein and curcumin for the treatment of melanoma.

## Data Availability

The raw data supporting the conclusions of this article will be made available by the authors on request.
